# Supplementary material for: Are carbon emissions trading and green financial instruments synergistic? -Comprehensive quantitative research based on content analysis
Source: PLoS One. 2024 Mar 7;19(3):e0298601. doi: 10.1371/journal.pone.0298601 (PMC10919601; doi:10.1371/journal.pone.0298601)
Supplement: S1 Data — (ZIP) [file pone.0298601.s001.zip › Data/地级市Z_F工作报告.zip!/║╙▒▒/▓╫╓▌╩╨/└┤╫╘╣1⁄2╓┌║┼í╛┬φ┐╦╩2╛▌═°í┐_2012.docx]

各位代表：

现在，我代表沧州市人民政府向大会作工作报告，请予审议，并请各位政协委员和其他列席会议的同志提出意见。

一、2011年工作回顾

2011年是实施“十二五”规划的第一年，也是我市建设沿海强市进程中重要的一年。面对复杂严峻的国内外经济形势，我们在省委、省政府和市委的正确领导下，认真贯彻落实科学发展观，开拓进取，奋力拼搏，大力推进沿海强市、和谐沧州建设，全市经济社会实现平稳较快发展，较好地完成了市十二届人大四次会议确定的各项任务目标，实现了“十二五”良好开局。

经济保持较快较好增长。全市地区生产总值完成2600亿元，增长12.3%。一、二、三次产业分别完成增加值295.8亿元、1358.7亿元和945.5亿元，增长5.3%、13.4%和12.8%。完成全社会固定资产投资1598亿元，增长26.2%。全部财政收入和一般预算收入分别突破300亿元和100亿元大关，全部财政收入完成328.5亿元，其中一般预算收入完成116.5亿元，分别比上年增长21.1%和27.6%。城市居民人均可支配收入18375元，农民人均纯收入6514元，分别增长14%和17.8%。全社会消费品零售总额674亿元，增长17.7%。

沿海经济快速发展。渤海新区发展实现新突破，沿海“一区一县”完成地区生产总值439.4亿元，增长18.3%，高于全市平均增速6个百分点；完成固定资产投资420.5亿元，增长32.1%；完成全部财政收入73.6亿元，其中一般预算收入20亿元，分别增长35.1%和35.7%。港口建设取得新进展，沿海基础设施进一步完善。黄骅港总吞吐量达到1.16亿吨，首次跨入亿吨大港行列。综合大港建设取得重大突破，争取交通运输部港口建设补助资金12.15亿元，20万吨级航道北防波堤、粮油码头等项目顺利推进，设计能力40万标箱的集装箱码头建成开航，港口对产业的拉动和服务功能进一步增强。沿海高速公路沧州至天津段建成通车，实现了黄骅港、天津港、唐山港、秦皇岛港的快速连接，有力地推动了我市沿海地区的开放、开发。中疏港路西延、205和307国道改造顺利通车，进一步完善了渤海新区的路网体系。产业聚集步伐进一步加快，旭阳化工40万吨己内酰胺、北汽40万辆整车等一批重大产业支撑项目开工建设，达力普特型铸锻、伦特150万吨沥青等项目竣工投产。河北农大渤海校区建成招生，北京中医药大学落户渤海新区，黄骅新城建设迈出实质性步伐。

重点项目建设扎实推进。全市投资亿元以上在建和竣工项目588个，比上年增加95个，总投资3754亿元，完成投资983亿元。其中投资5亿元以上项目165个，比上年增加78个。列入省重点项目101个，完成投资384亿元。华北石化千万吨炼油完成了全部审批手续，进入实质性建设阶段；中海油中捷石化、中石化沧州分公司炼油质量升级改造项目分别获得中海油、中石化批准，任丘百万吨乙烯项目列入国家规划，为我市建设石油化工强市奠定了坚实基础。泰恒特钢100万吨不锈钢、东塑新能源材料工业园等294个亿元以上项目开工建设；中钢8万吨镍铁、宏润重工5万吨压力机、沧州大化10万吨TDI等117个亿元以上项目顺利推进；河北万邦重型机械、东光百利高阻隔膜等172个亿元以上项目竣工投产，新增销售收入1500多亿元。

中心城区经济发展呈现新气象。市政府出台了《关于加快沧州中心城区经济发展的意见》，成立了中心城区经济发展领导小组。沧东经济开发区被批准为省级开发区，沧州高新区跨入省级高新区行列。鑫源泰巨龙钢管、华油飞达钻机等项目竣工投产；中能环科高温凝结水处理装备、拓达管业钢管生产线、泰大国际、彩龙商贸广场、颐和新世界、锦绣天地等项目开工建设；大连宏海新能源风电光电、航天神舟太阳能光热产业园、百阳集团电动汽车等项目落户沧州。

城镇面貌发生新变化。中心城区一批重点城建项目建成投用，京沪高铁站及站前广场、名人植物园等成为我市的新亮点。京沪高铁站综合楼被评为“河北省十佳公共建筑”，迎宾大道被评为“河北省十佳景观大道”，名人植物园、肃宁县人民公园被评为“河北省十佳公园”。沧州体育场、图书馆、博物馆、城乡规划馆、市民服务中心主体封顶，管道大厦、移动通讯枢纽楼等21个企业总部和沧州游泳馆正式开工。西外环一期工程、贵州大道、吉林大道竣工通车。新建改造供水、供热、燃气管网64.3公里，新增集中供热面积118万平方米。京沪铁路维明路立交桥改建工程和运河景观带整治工程正式启动。皇家壹里、兰亭苑、东方世纪城等精品住宅小区加快推进。新增绿地面积1348.5亩，建成区绿化覆盖率达到43.7%。市区空气质量二级以上天数达到345天，一级天数比上年增加41天。县城“十个一”工程共实施建设项目142个，完成投资214.9亿元。泊头市、肃宁县成功创建省级园林城市。全市城镇化率达到43%，比上年提高1个百分点。

工业聚集区和开发区建设取得大进展。市政府出台了《关于加快工业聚集区发展的实施意见》。经过积极争取，省政府新批准我市省级工业聚集区、开发区11个，进一步为产业发展搭建起坚实平台。全市省级以上工业聚集区、开发区总数达到18个，入驻企业4011家，完成税收124亿元，增长18%；完成固定资产投资455亿元，增长40%；规模以上工业完成增加值400亿元，增长29.3%；小洋人工业新城、孟村志航钢管等投资10亿元以上项目达到45个。沧州临港经济技术开发区、任丘开发区、盐山管道管件工业聚集区主营业务收入超过百亿元。

工业经济提质增效。规模以上工业完成增加值首次突破千亿元，达到1041亿元，实现利税448亿元，分别增长16.8%和23%；新增规模以上工业企业112家，总数达到1454家。销售收入超100亿元的企业达到5家，比上年增加2家。渤海重工、沧海管件等13家企业被认定为高新技术企业，高新技术产业完成增加值125亿元，增长30%，占规模以上工业增加值的比重达到12.1%。大力实施“1+1”工程，启动亿元以上技改项目150个，总投资742亿元。深入实施品牌带动战略，“海浩”、“南风”、“妙恋”、“恒通图形”被评为中国驰名商标。省“双三十”和“8755”减排工程扎实推进，节能减排和环境保护取得新成效。

农业生产再登新台阶。粮食总产首次突破百亿斤，达到501万吨，连续八年丰产丰收。棉花、蔬菜、果品产量较快增长，肉、蛋、奶产量不断提高。培育省级以上畜禽养殖标准化示范场49个，认定无公害蔬菜基地2个、畜产品产地16个。新增农民专业合作社545家，总数达到1890家，辐射带动农户63万户。市级以上农业产业化龙头企业达到390家，比上年增加51家，农业产业化经营率51%。完成植树造林21.8万亩。抓住国家支持水利改革发展的机遇，深入实施跨流域调水和“十百千”工程，为大浪淀水库、杨埕水库引蓄黄河水1.3亿立方米，东光观州湖水库建成蓄水，肃宁水库开工建设，全市新增蓄水能力2300万立方米。

县域经济实力不断壮大。管道装备、五金机电、汽车模具、裘皮服装、电线电缆等特色产业发展跨上新台阶，南皮、青县分别被授予“中国五金机电产品生产基地”、“中国电子机箱制造基地”称号。盐山管道装备制造、孟村弯头管件、黄骅化工、南皮五金机电、献县扣件铸造、河间电线电缆等6个特色产业集群销售收入超百亿元。引进了中航重工、香港南华集团、北京三友集团等一批大企业、大集团，东光化工、孟村华洋、河间新宝丰、肃宁华斯等龙头骨干企业规模和层次不断提高，对县域经济发展的支撑能力进一步增强。放手发展民营经济，全市民营经济单位总数达到20.4万个，完成营业收入8175亿元，实现增加值1600亿元，分别增长39.8%和13.3%，民营经济已成为县域经济发展的主体力量。全市所有县（市）财政收入全部超过3亿元，其中6个县（市）超过10亿元。

对外开放迈出新步伐。全市实际利用外资完成2.92亿美元，增长25.7%，引进省外资金368亿元，增长97.8%。进出口总额完成21.5亿美元，增长28%，其中出口18.8亿美元，增长35%。成功举办了第二届中国（沧州）国际塑料展，签约项目21个，总投资612.8亿元。全市域内银行贷款余额1088亿元，首次突破千亿元。华夏银行入驻沧州，浦发银行、兴业银行沧州分行正在加紧筹建。新增保险机构3家，小额贷款公司6家、担保公司15家。沧兴商砼、龙马钢管、双剑机械等7家企业在天交所渤海股权交易中心挂牌上市，新增上市企业数量全省第一。我市连续三年被省政府评为“金融生态市”。

保障改善民生力度加大。全市开工建设保障性住房38971套，完成省下达任务的121%。新增国家级新型农村养老保险和城镇居民养老保险双项试点县8个，总数达到13个。城镇职工医保覆盖率达到95.6%，新农合参合率达到94%，城乡居民养老保险覆盖面达到71.8%。市本级城镇职工和市区城镇居民医保基金最高支付限额由5万元提高到7万元，在校学生和未成年居民由每人每年7万元提高到10万元。医改工作扎实推进，全市170个政府举办的乡镇卫生院全部实现基本药物零差率销售，人均基本公共卫生服务经费标准从18元提高到25元。基层医疗卫生体系不断完善，新建县级医院7个、中心乡镇卫生院7个、村卫生室83个，中心城区社区卫生服务街道覆盖率达到95%以上。新增城镇就业5.5万人，下岗失业人员实现再就业2.5万人，农村劳动力转移就业14.8万人，城镇登记失业率控制在3.8%以内，被省政府评为劳务经济先进市。大力实施饮水安全工程，解决了80万人的饮水安全问题。

各项社会事业全面进步。教育事业快速发展，学前幼儿入园率达到91.5%，全市高中阶段毛入学率达到70%，主城区实现基本普及高中阶段教育，并通过省政府评估验收。化解义务教育债务3亿多元，累计改造中小学校336所，消灭D级危房81.2万平方米。大力发展文化体育事业，群艺馆、图书馆、博物馆全部免费开放，西路梆子和六合拳入选国家级非物质文化遗产名录，成功举办了第十三届中国·吴桥国际杂技艺术节沧州会场。安全生产和社会治安形势保持稳定，食品药品安全监管进一步加强。用地环境安全有序，连续三年被国土资源部评为“双保工程”成效显著单位。全面落实双拥优抚安置政策，军民团结、军政团结的良好局面进一步巩固。民族宗教、外事侨务、妇女儿童、爱国卫生、应急保障、人民防空、防震减灾、广播电视、气象、测绘、档案、老龄、残疾人等事业都取得了新的成绩。

政府自身建设不断加强。高度重视向市人大常委会报告工作，自觉接受人大法律监督、政协民主监督和社会监督，办理市人大代表建议154件，市政协提案266件，办复率100%。大力弘扬求真务实、真抓实干的工作作风，狠抓各项工作任务的落实。加强机关效能建设，简化审批手续，提高办事效率，进一步优化发展环境。在市政府系统开展了“爱沧州、做贡献、干成事、出亮点”活动，强化广大干部职工的责任意识、服务意识、奉献意识，大力营造风清气正、干事创业的浓厚氛围。认真落实党风廉政建设责任制，加大审计、监察工作力度，严肃查处违法违纪案件。积极推进依法行政，严格依照法定权限和程序履行职责，政府工作决策程序进一步规范，透明度和公信力不断提高。

各位代表，过去的一年，在国际市场需求减弱和国内宏观经济政策偏紧的形势下，全市上下团结拼搏，共克时艰，抢抓机遇，发挥优势，开拓创新，推动沧州经济社会实现了平稳较快发展，人民生活水平有了新的提高。这些成绩的取得，是党中央、国务院和省委、省政府、中共沧州市委正确领导的结果，是全市720万人民共同奋斗的结果，凝聚着全市人民的心血和汗水。在此，我代表沧州市人民政府，向辛勤工作在各条战线上的全市各族人民，向给予我们大力支持的人大代表、政协委员和各民主党派、工商联、无党派人士，向大力支持我们工作的离退休老干部、驻沧各单位、人民解放军和武警官兵，向所有关心、支持沧州发展的同志们、朋友们，致以崇高的敬意和衷心的感谢！

在肯定成绩的同时，我们也清醒地看到，我市经济社会发展中还存在一些亟待解决的矛盾和问题：沿海经济刚刚起步，发展的任务依然繁重；中心城区经济仍然不强；一般预算收入占全部财政收入比重偏低；资金、土地、水资源等制约和影响发展的瓶颈问题还比较突出；保障和改善民生的任务十分繁重，政府自身建设仍需进一步加强。对这些问题，我们将采取更加有力的措施，认真加以解决。

各位代表，去年，京沪高铁通车，使我市进入了首都一小时交通圈；沿海高速公路全线贯通，进一步增强了我市沿海开发的优势；港口码头功能的进一步完善，吞吐量超过亿吨，港口对临港产业和全市经济发展的拉动作用明显增强；一大批重大项目的开工建设，进一步奠定了我们建设沿海强市的基础；国务院批准实施《河北沿海地区发展规划》，给我们带来了前所未有的历史机遇。我们一定要倍加珍惜来之不易的好形势，全力抢抓千载难逢的好机遇，团结一心，奋力拼搏，努力开创沧州经济社会发展的新局面！

二、2012年政府工作的指导思想和主要预期目标

今年是全面实施《河北沿海地区发展规划》、推进沿海强市建设的关键一年。做好今年的政府工作，对于加快沿海强市建设、全面完成“十二五”规划至关重要。

综合研判国内外经济形势，今年我们既面临许多严峻挑战，也面临着发展的重大机遇。当前，世界经济形势十分严峻复杂，世界经济复苏的不稳定性、不确定性上升，国际经济环境难以明显好转，国际市场仍将持续低迷。中央实施稳健的货币政策，信贷不会有太大的增长。今年的宏观经济形势，势必对我市经济发展产生很大的影响。在理性分析困难的同时，我们更要看到我市面临的机遇和优势：一是《河北沿海地区发展规划》上升为国家战略，我市的战略地位和竞争优势快速提升，国家和省重点支持我市发展，国内外投资者越来越关注我市，为我们带来加快发展的重大机遇。二是我市的港口优势、京津一小时交通圈优势、土地优势、较低的劳动力成本优势，为制造业和服务业提供了发展的比较优势，对外企、央企、大型民营企业、京津产业转移、南资北移产生了很强的吸引力。三是今年中央继续实施积极的财政政策和支持实体经济发展政策，适当增加中央预算内投资，并实行结构性减税，引导各种资金投向实体经济，为我市加快发展工业和服务业带来了机遇。我们一定要抓住机遇，发挥优势，把困难估计得更充分一些，把措施考虑得更周密一些，牢牢把握经济社会发展的主动权。

今年政府工作的指导思想是：以邓小平理论和“三个代表”重要思想为指导，深入贯彻落实科学发展观，全面落实省、市第八次党代会精神，坚持“稳中求快、稳中求好”的工作总基调，大力实施“从大运河走向渤海湾”发展战略，全面实施沿海发展规划，强力推进沿海经济、中心城区经济、县域经济快速发展，全面发展社会各项事业，切实保障和改善民生，努力实现全市经济社会更好更快更大发展。

主要预期目标是：全市地区生产总值增长11%以上；全部财政收入增长15%，其中一般预算收入增长16%；全社会固定资产投资增长20%；规模以上工业增加值增长15%；社会消费品零售总额增长17%，实际利用外资增长20%，出口总值增长15%；城镇居民人均可支配收入增长10%，农民人均纯收入增长10%；人口自然增长率控制在7.5‰以内；万元地区生产总值综合能耗下降3.66%。

实现上述任务目标，要牢牢把握六个原则：第一，把握稳中求快、稳中求好这一总基调。稳，就是要保持经济平稳较快增长，保持社会大局稳定；快，就是要抓住难得的发展机遇，加快发展，真正成为全省的重要增长极；好，就是要提高经济发展的质量效益，推进经济发展方式转变，优化经济结构，实现全面协调可持续发展。稳是基础，快和好是目标，要在稳的基础上，努力实现又好又快发展。第二，强化机遇意识，切实增强发展的紧迫感、责任感。紧紧抓住《河北沿海地区发展规划》上升为国家战略等一系列重大机遇，认真研究上级政策，最大限度地用足用好政策，把政策优势加速转化成为竞争优势、发展优势，以强烈的事业心、责任感和只争朝夕的精神，一心一意谋发展，凝神聚力抓落实，努力开创沿海强市建设的新局面。第三，坚持统筹兼顾，抓重点、破难点、出亮点。面对繁重的发展任务，必须紧紧抓住事关全局的重点工作不放松，牢牢扭住主要矛盾和关键环节求突破，以重点、难点问题的突破带动活跃全局。第四，坚持民生为本，切实保障和改善民生。我们发展的根本目的就是要不断提高人民群众的生活水平。必须牢记使命，时刻把人民群众的安危冷暖放在心上，把解决人民群众反映强烈的问题抓在手上，在加快发展经济的同时，进一步健全和完善社会保障体系，努力办成一批受益面广、群众满意的民生工程，让人民群众共享改革发展的成果。第五，坚持开拓创新，用新思路、新办法破解发展瓶颈。坚持以创新的思维、创新的理念寻求破解之策，以创新的举措、创新的方法突破瓶颈制约，尤其是要着重解决好发展中面临的用水、用地、资金、技术、人才等多重瓶颈制约，为全市经济社会又好又快发展提供有力保障。第六，大力改进工作作风，努力干事、干成事。求真务实、真抓实干的工作作风是事业成功的前提和基础。必须顺应时代发展要求，牢记执政为民理念，爱岗敬业，拼搏奉献，以饱满的热情、昂扬的斗志，敢于争先，敢啃“硬骨头”，努力干成一批关系沧州发展的大事、实事。

各位代表，在建设沿海强市的征程中，我们会面临一些困难和问题，但我们更面临着前所未有的发展机遇。《河北沿海地区发展规划》上升为国家战略，省委、省政府高度重视沧州的发展，沧州吸引了全国各行各业投资者的目光，沧州前所未有地受到广泛关注。只要我们坚持发展不动摇，抢抓机遇，发挥优势，奋力拼搏，就一定会一步一个脚印地把沧州的发展不断推上新的台阶！

三、奋力开创经济社会发展新局面

把握稳中求快、稳中求好的总基调，以建设沿海强市为目标，突出重点、统筹兼顾，着力做好以下十个方面的工作：

（一）抢抓《河北沿海地区发展规划》实施的机遇。《河北沿海地区发展规划》范围包括全市所有县（市、区），涵盖了工业、农业、服务业、城乡发展、基础设施、生态环境保护，以及社会事业和民生建设等方方面面，是指导我市发展的全局性、战略性规划。我们要把学习好、宣传好、落实好《规划》作为一项重要任务，以《规划》为指导，推动全市经济社会发展稳中求快、稳中求好。一是深入学习研究规划。加强对规划总体要求、任务目标、思路举措、产业政策、优惠条件等方面的学习研究，结合工作实际，深入研究推动规划、落实规划的切入点和突破口，谋划一批落实《规划》的重大项目、重大工程和重点工作。二是加大宣传推介力度。大力宣传我市的区位、交通、港口、土地资源、低成本等综合优势，让更多企业家、投资者了解规划、了解沧州，增强投资沧州、发展沧州的信心。三是加强与中央部委和省直部门的沟通协调。落实重大事项、重大项目跑办责任制，紧紧围绕项目审批、贷款融资、用地指标等方面，跑部进京、跑省进厅，争取更多政策、资金和项目支持。突出抓好项目跟踪、跑办，确保重大建设项目早审批、早开工、早投产。

（二）奋力推进沿海经济发展实现新突破。省第八次党代会提出“举全省之力打造曹妃甸新区和渤海新区两大增长极”。要以贯彻落实省第八次党代会精神，全面实施《河北沿海地区发展规划》为契机，奋力推进沿海地区开发开放，加快渤海新区建设步伐，推动沿海经济发展再上新水平。一是进一步完善港口功能。充分发挥散杂货码头和集装箱码头功能，拓展腹地，广辟货源，增加吞吐量，进一步拉动临港产业和全市经济发展。加快推进20万吨级铁矿石码头和20万吨级航道建设，继续完善集装箱码头，力争设计通过能力达到90万标箱。二是进一步推动临港产业发展。围绕建设石油化工基地、化工新材料基地、装备制造基地、特钢基地、电力能源基地，发挥海运成本低、土地资源丰富等优势，推动石油化工、装备制造、冶金新材料等产业向沿海聚集，谋划建设一批产业配套衔接、上下游一体的新型工业化项目。重点抓好金牛化工40万吨PVC、旭阳40万吨己内酰胺、北汽40万辆整车、中国一重冷轧板等重大项目。加快推进冀中南工业聚集区、冶金装备园区、物流产业聚集区、电力能源聚集区等各类园区建设，打造特色鲜明、竞争优势明显的临港产业集群。三是大力推进黄骅新城建设。加快新城区企业总部、北京中医药大学东方学院、国际文化创意广场、博海公园等重点工程，以及商贸餐饮、仓储物流等服务设施建设，增强对要素资源和优质产业的吸引力。

（三）狠抓重点项目建设。重点项目建设是我市加快发展的最重要途径，也是调结构、转方式的主要办法，要坚定不移地狠抓重点项目建设，紧紧围绕新型工业化项目、服务业项目，以及新能源、新材料等战略性新兴产业项目，集中力量建设一批立市兴县的重大产业项目。一是抓项目开工。把项目开工率作为项目建设的重要指标，千方百计推进项目前期工作，重点协调解决好项目审批、资金筹措、用地指标等问题，力促谋划项目早签约，前期项目早审批，批复项目早开工，确保中海油中捷石化炼油质量升级改造、渤海新区热电、润恒农副产品冷链物流、泊头恒盛舰船专用泵等200个重点项目开工建设。二是抓项目投产达效。对投资规模大、带动能力强的重点项目加强协调调度，解决实际问题，确保沧州大化10万吨TDI、福道冶金12万吨轧辊、泊头安吉宏业汽车加热器、任丘热电等100个重大项目投产达效。三是抓项目谋划。着力提高项目谋划的能力和水平，重点围绕发挥油气资源、延伸产业链条、壮大石化产业搞好项目谋划，努力把沧州打造成为绿色、安全、国际石油化工城。围绕发挥特色产业优势，以管道装备、机械制造、纺织服装、食品加工等产业为重点，积极谋划一批能够壮大产业规模、提升产业层次的大项目、好项目。

（四）加快中心城区经济发展。一是进一步强化发展中心城区经济的意识。中心城区具有较完善的基础设施和较好的服务产业发展功能，对产业有较强的承载力，中心城区经济必须要发展得更快更好一些。同时，只有中心城区产业发展、实力增强，才能更好地服务和拉动全市经济发展，才能实现整个市域经济较快、可持续发展。坚持一手抓工业，一手抓服务业，以工业发展扩大就业、聚集人口、扩张城市规模、带动服务业发展，以服务业发展促进产业聚集、提升工业发展水平，形成“二产带三产，三产促二产”的互动发展格局。二是加快三个园区发展。以沧州开发区、高新区、沧东开发区为平台，充分发挥中心城市要素资源吸纳能力强、配套服务设施完备的优势，积极引进战略投资者，谋划建设一批投资规模大、技术水平高、无污染的高新技术项目和现代装备制造业项目。重点抓好河北拓达、巨龙钢管、东塑新能源等一批重点项目建设，力争中国航天科技集团太阳能光热产业园、台商工业园、百阳集团电动汽车、宏海新能源等一批重点项目开工建设，确保三个开发区工业增加值、主营业务收入、税收增长30%以上。三是积极发展服务业，全力打造“七大服务中心”。积极发展金融保险、研发设计、服务外包、仓储物流、会展经济等新兴服务业。突出抓好小南门、火车站广场等传统商业区升级改造，推进泰大国际、锦绣天地、彩龙商贸广场等一批城市综合体项目建设，引进一批国内外知名的大型商贸物流企业，培育发展一批大型商贸物流企业集团，尽快使中心城区成为全市的金融服务中心、商贸服务中心、物流配送服务中心、科技研发和技术服务中心、社会中介服务中心、消费服务中心和公共服务中心，全面提高服务业发展水平，提高对经济增长的贡献率。

（五）着力推进城镇建设再上新水平。以完善配套设施、提升城市功能、聚集优质产业、强化精细管理为目标，推动城镇建设上水平。一是加快中心城区重点城建工程建设。确保体育场、图书馆、博物馆、城乡规划馆、市民服务中心等项目建成投用。加快推进管业大厦、供排水大厦、移动通讯枢纽楼等21个企业总部项目建设，确保完成形象进度。加快游泳馆建设，加快京沪铁路维明路立交桥改建。按照保护、恢复、开发的思路，推进运河市区示范段开发整治工程，塑造运河文化特色，打造靓丽的城市景观。加快管网配套工程建设，新建供排水管网24公里，供热管网13公里。扎实推进运东热电联产项目，力争尽快实现中心城区集中供热全覆盖。大力实施绿化、美化、亮化工程，加快千童公园建设，开工建设滨河公园，着力抓好开元大道等4条街道景观整治和11条街道景点的绿化工程，新建街头游园15个。积极稳妥地推进城中村和旧城区改造，进一步改善群众居住条件。二是切实加强城市管理。整合城市管理资源，理顺体制机制，加强社区建设，延伸城管触角，发挥数字化平台作用，提高城市管理水平。实施市民素质提升工程，深入开展城市环境综合治理活动，着力解决脏、乱、堵问题，打造环境优美、规范有序、便捷舒适、文明和谐的城市环境。三是继续加快县城和重点镇建设。以道路、供水、供电、供热、商贸流通、教育文化等设施建设为重点，加快完善县城功能，扩大县城规模，提高对要素资源的吸纳能力。大力推进任丘、泊头、河间等县级市向中等城市迈进。坚持高起点规划、高标准建设，加快小城镇建设步伐，在中心城区周边和各县（市）着力培育一批人口超3万、5万的重点镇，打造一批中心城区和县城的卫星镇。

（六）继续加大工业聚集区和开发区建设力度。工业聚集区和开发区是实施工业强市战略、推进城市化的重要平台，也是工业经济集约集群发展的重要载体。要推动企业、项目向工业聚集区和开发区聚集，为市域经济发展构筑支撑。工业聚集区和开发区固定资产投资、主营业务收入、增加值、税收要增长25%以上。一是加大招商引资和项目建设力度。实行招商目标管理责任制，努力做到人人都有招商任务，人人都能引来项目。制定园区综合考核评价办法，建立动态管理和扶强促优机制，抓好项目跟踪落实，跑前期、促开工、求达效，壮大工业聚集区和开发区规模，提升发展层次，培育一批主营业务收入超100亿元乃至500亿元的大园区。二是完善基础设施，优化发展环境。按照适度超前的原则，推进工业聚集区和开发区水、电、路、气、讯，以及污水处理、垃圾处理等基础设施建设，为招商引资、项目入区和企业发展提供良好的“硬环境”。进一步转变工作作风，简化办事流程，提高工作效率，真心实意地为投资者服务，为企业发展服务，创造良好的“软环境”。三是发挥政策优势，促进园区建设提速升级。用足用好支持工业聚集区、开发区发展的政策措施，在土地、资金、电力、交通、人才引进、要素资源配置等方面加大扶持力度，促进工业聚集区和开发区建设提速升级。

（七）坚定不移地实施工业强市战略。实施工业强市战略，加快新型工业化步伐，是推动沧州经济社会又好又快发展的必然选择，也是沿海强市建设的必由之路，必须坚定不移强力推进。一是着力抓好规模企业发展。重点培育一批销售收入超百亿元、超五百亿元的大企业、大公司、大集团，在各县（市、区）培育一批销售收入超十亿元、超五十亿元的大企业。加大扶持力度，促进企业做大做强。二是抓好现有企业发展。招商引资、引进新企业是加快工业发展的一条重要路径，把现有企业做大做强同样也是建设工业强市的重大举措。大力开展标杆管理活动，推动企业瞄准国内外本行业规模大、效益好、技术水平高的企业，开展对标学习活动，在管理、技术开发、市场营销等方面迅速提高企业的水平，增强企业的市场竞争力。热情服务，主动解难，扶持企业发展。三是加快企业技术改造。大力实施“1+1工程”，用政策鼓励每个企业每年都要实施一个技改项目，研发一个新产品。着力抓好渤海重工、河北信诺立兴、那瑞化学科技等100家重点企业的技术改造，促进企业提档升级。充分发挥工业企业技术改造专项资金的激励和导向作用，引导企业加大科技研发投入，争取引进、嫁接改造一批高端项目，力争技术改造对工业增长的贡献率达到40%以上。大力实施品牌战略，争创中国驰名商标，打造一批具有较高知名度和较强竞争力的品牌和产品。四是抓好工业运行监测，保障企业满负荷生产。加强工业运行监测，及时协调解决企业发展中遇到的问题，确保全市工业健康快速发展。突出抓好生产要素调节，在保障重点企业煤电油气运等生产要素供应的同时，在企业用工、人才培训、融资贷款、项目用地、市场开拓等方面，强化跟踪服务，为企业生产经营创造一流的环境。五是推进节能减排，确保完成年度任务目标。进一步完善目标管理责任制和目标考核机制，严格落实问责制和“一票否决制”，确保各项措施不折不扣地落实到位。严把项目准入关、环保审批关，从源头上杜绝“两高一低”项目上马。支持企业采用新工艺、新材料、新设备，加快淘汰落后产能。着力抓好重点领域、重点行业、重点企业的节能减排工作。积极推进污水处理厂和垃圾处理厂建设，确保如期建成，发挥效益。深入开展循环经济示范园区、示范企业创建活动，鼓励重点园区、重点企业大力发展循环经济、低碳经济。

（八）扎实做好“三农”工作。农业、农村、农民工作始终是我们工作的重点。继续加大强农、惠农、富农政策支持力度，努力促进农业增效、农民增收、农村发展。一是积极发展现代农业。在稳定粮食生产的基础上，稳步推进规模经营，按照标准化、规模化、集约化、现代化的目标，瞄准京津市场，在特色上做文章，在高效上找出路，大力推进畜牧水产养殖基地、果蔬种植基地、优质棉花基地和高标准农田示范县建设。继续实施科教兴农战略，开展农业科技攻关，加大种植、养殖新技术、新品种的引进推广力度，提高农业发展的质量和效益。二是大力推进农业产业化经营。加强农村合作组织建设，大力培育带动能力强的龙头企业，打造叫得响的农产品品牌。加大对农业产业化龙头企业的扶持力度，发挥龙头企业引领带动作用，加快农业产业化进程，提高农业综合效益。重点抓好河间国欣棉花、献县沧华园枣业、青县齐发蔬菜、肃宁绿苑蔬菜等100家示范性农村专业合作社，力争每个县（市）拥有1-3个标准农业产业示范园。三是不断完善农村基础设施和农村公共服务。抓住国家大力发展农业、农村基础设施的机遇，积极争取农田水利、电力供应、农村公路、集中饮水等基础设施建设项目。以农村义务教育、中等职业教育、新型农村合作医疗和新型农村社会养老保险制度改革为重点，推进公共服务和社会保障向农村延伸，努力改善农民群众的生产生活条件。加大农村道路、桥梁、供电设施建设力度，实施610个村的电网改造升级工程，新建改建农村公路500公里，改造农村桥梁22座。四是千方百计增加农民收入。认真贯彻落实国家粮食直补、良种补贴、农机具购置补贴等各项惠农政策，不断加大财政支农、惠农力度。抓好农村劳动力技能培训和劳务输出，有序引导农民进城务工，积极发展农村二、三产业，提高农村人口工资性收入。全面落实国家和省新十年扶贫开发纲要，全力打好扶贫开发攻坚战，让更多贫困人口尽快脱贫致富。

（九）进一步推进改革开放。在市场经济条件下，区域经济加快发展、跨越发展，关键靠开放。能不能创造良好的投资环境，吸引生产要素流向一个地方，重要的举措是改革。要利用好我市的诸多优势，进一步加快改革步伐，推进全方位开放，争取更多的项目、资金、人才涌入我市。一是大力开展招商引资。在全市形成人人招商、全员招商的开放格局。充分发挥我市环京津、沿渤海优势，开展全方位招商引资活动，努力引进一批大型央企、民企和外资企业。内资瞄准京津，面向“长三角”、“珠三角”，主抓京津产业转移和南资北移；外资在巩固港澳台、日韩和欧美的同时，不断拓展新的招商区域。加强与世界500强、国内500强大企业的战略合作，努力把沧州打造成为京津产业的转移基地、总部经济的生产加工基地和外资企业的聚集基地。二是着力引进高端人才和科技成果。进一步扩大与京津经济技术合作的范围和领域，借助京津科技优势，引进资金、技术、人才，加强生产力促进中心建设，发展一批科技成果中试基地，促进科技成果向现实生产力转化。三是大力发展外向型经济。加大对重点出口企业的扶持力度，重点抓好石油钻采设备、管道装备、精细化工、裘皮服装等出口基地建设，培育特色出口品牌，提升企业参与国际市场竞争的能力，不断扩大出口规模，增强外贸出口对经济增长的拉动作用。四是加快体制机制创新。深化行政管理体制改革，减少和规范行政审批事项和工作流程，建立充满活力、富有效率、更加开放、有利于科学发展的体制机制。大力扶持民营经济发展，全面落实鼓励民营经济发展的政策措施。积极推进林权制度、投融资体制和社会管理体制等综合配套改革。深化医药卫生体制改革，全面完成基层医疗卫生机构综合改革任务，稳步推进公立医院改革，巩固和扩大医改成果，逐步解决群众看病难、看病贵的问题。

（十）全面加强和谐沧州建设。坚持把改善民生、提高人民群众生活水平，作为政府工作的首要职责，进一步加大财政投入力度，扩大公共服务范围，让人民群众得到更多实惠。一是推进就业再就业工程。积极实施就业援助工程，重点针对零就业家庭、残疾人、低保对象等就业困难群体和高校毕业生，大力开发就业岗位，努力提高就业水平。鼓励有创业能力的人员自主创业、自谋职业，以创业带动就业，确保城镇新增就业4.95万人，城镇登记失业率控制在4.5%以内。二是着力提高社会保障水平。抓好城乡居民养老保险试点工作，在13个县（市、区）的基础上，力争今年实现全覆盖。积极稳妥地推进养老、医疗、工伤、失业、生育“五险”扩面提升，城镇居民医保、新农合补助标准提高到每人每年240元，城乡居民最低生活保障人均补差水平分别提高到每人每月240元和110元。完善社会保障“一卡通”制度，逐步建立“五险合一”工作机制。三是加强保障性住房建设。进一步完善保障性住房建设管理模式，抓好土地供应、资金筹措和质量监管等重要环节，确保完成26700套保障性住房建设任务。按照“公开、公正、透明”的原则，健全保障性住房分配制度，着力解决低收入群体住房困难问题。四是大力发展教育、科技事业。实施主城区教育信息化工程，推进学前教育三年行动计划，加快义务教育学校标准化建设，逐步普及高中阶段教育。积极推进一中迁建、二中学生公寓、十四中新建教学楼、工贸学校一期工程、沧州师院二期、河北工专新校区等项目建设。深入实施校舍安全工程，加快农村学校改造、农村幼儿园新建和改扩建等专项工程建设。结合产业发展需要，组织一批重点企业与知名院校、科研单位开展产学研对接活动，建立一批技术创新战略联盟和科技成果转化基地。五是繁荣文化体育事业。开工建设市妇女儿童和青少年文化活动中心。大力发展文化产业，谋划推进一批文化产业项目，支持文化精品创作，提升沧州运河文化、渤海文化的凝聚力、影响力和竞争力，推动文化大市向文化强市转变。继续加强体育公共设施建设，广泛开展群众性全民健身活动，积极做好第十四届省运会各项筹备工作。六是认真做好医疗卫生和计划生育工作。积极推进和完善国家基本药物制度，进一步加强乡镇卫生院和村卫生室、城市社区卫生服务中心建设，完善基层医疗卫生服务体系，促进公共卫生服务均等化。加强人口和计划生育工作，继续稳定低生育水平，全面推进优生优育，下大力治理出生人口性别比偏高问题。七是着力解决饮水安全问题。启动南水北调中线工程，继续完善引黄线路，深入实施饮水安全工程，加大财政投入力度，集中力量解决60万人的饮水安全问题。八是加强安全生产监管。严格落实安全生产责任制，加强对重点企业、重点行业、重点领域，以及人员密集场所的监管，深入开展隐患排查治理和专项整治活动，坚决遏制重特大安全生产事故发生。九是切实抓好食品药品监管。认真开展食品药品安全专项整治活动，加强对地沟油、添加剂、熟肉制品、枣制品等重点行业、重点产品的监管，加大对制售假冒伪劣行为的查处打击力度，让全市人民群众买得放心、吃得安心。十是维护社会和谐稳定。加强和创新社会管理，推进公共服务均等化，着力抓好城乡社区建设，规范发展社会组织，努力实现社会管理与社会服务有机统一。健全完善利益诉求表达、矛盾纠纷排查化解、社会治安防控等工作机制，加强法制宣传教育，坚持依法依规办事，促进社会公平正义。深入开展“打四黑、除四害”专项行动，消除损害群众利益和影响社会稳定的各种隐患，努力构建“大维稳”格局，积极打造平安沧州、和谐沧州。

高度重视发展老龄事业，依法保障妇女儿童和未成年人权益，关心支持残疾人事业。全面贯彻党的民族宗教政策，积极扶持回族等少数民族群众发展经济，提高生活水平。支持工会、共青团、妇联、红十字会等人民团体发挥桥梁纽带作用。积极做好外事侨务、气象测绘、史志档案等工作。加强国防教育和国防后备力量建设，支持驻沧人民解放军、武警部队、预备役部队、民兵和人民防空建设，认真做好部队家属就业、子女上学等方面的工作，深入开展双拥共建活动，巩固军政军民团结的好局面。

推进沿海强市、和谐沧州建设，政府责任重大。各级政府必须进一步加强自身建设，转变职能，着力建设法治政府、责任政府、服务政府、创新政府、效能政府和廉洁政府。一是强化服务，一心为民。牢固树立人民至上的信念，全心全意为沧州人民服务。二是科学决策，依法行政。健全科学民主决策机制，严格按照法定权限和程序履行职责。自觉接受市人大及其常委会的法律监督和工作监督，接受市政协的民主监督，虚心听取各民主党派、工商联、无党派人士及社会各界的意见和建议，悉心倾听人民群众的意愿和呼声。三是廉洁勤政，干净干事。严格执行党风廉政建设责任制，深入推进惩治和预防腐败体系建设，切实做到勤政、为民、务实、清廉。四是求真务实，开拓创新。在政府系统深入开展“爱沧州、做贡献、干成事、出亮点”活动，动员市政府各部门、全体工作人员进一步增强发展的责任感、紧迫感，立足岗位“爱沧州”，奋发有为“做贡献”，奋力拼搏“干成事”，争先创优“出亮点”，勤奋工作，攻坚克难，努力干成一批在沧州发展史上有重大影响的大事、实事。

各位代表，沧州是我们可爱的家乡，把沧州建设得更加美好，是720万沧州人民的共同心愿。机遇前所未有，形势催人奋进。让我们在党中央、国务院和省委、省政府、中共沧州市委的正确领导下，鞠躬尽瘁，勤奋工作，为建设宜居宜业的现代化沿海新沧州而努力奋斗！
